# Psychological correlates of COVID safety protocol adherence among university students

**DOI:** 10.4314/gmj.v57i1.8

**Published:** 2023-01

**Authors:** Frances E Owusu-Ansah, Christian Amoah, Akua A Addae, Victoria DeGraft-Adjei, Addo Frimpong-Manso, John Appiah-Poku

**Affiliations:** 1 Department of Behavioural Sciences, Kwame Nkrumah University of Science and Technology, Department of Behavioural Sciences, School of Medicine and Dentistry, KNUST, Ghana; 2 Counseling Centre, Kwame Nkrumah University of Science and Technology, Ghana

**Keywords:** Psychological well-being, self-esteem, COVID-protocol adherence, perceived control, university students

## Abstract

**Background:**

The COVID-19 pandemic continues to be a global concern. Reports of insidious asymptomatic variants of the virus raise concerns about the safety of huge numbers of students on university campuses.

**Objective:**

The study aimed to delineate psychological correlates for students' adherence to safety protocols for appropriate context-specific coping intervention designs.

**Setting & Design:**

751 students from the various colleges of the KNUST were conveniently sampled for this cross-sectional survey.

**Measures:**

Psychological instruments with good psychometric properties (DASS-21; Rosenberg Self-Esteem Scale and Perceived Control Scales) were used in addition to demographics and questions on COVID safety protocol adherence.

**Results:**

Self-esteem positively correlated with perceived control (r = 0.40, p<0.001) and COVID adherence (r = 0.16, p<0.001); but negatively correlated with psychological distress (r = -0.44 p<0.001). Greater perceived control was associated with lower psychological distress (r = -0.20 p<0.001) and greater adherence to safety protocols (r = 0.24 p<0.001). Protocol adherence was regressed on psychological distress, self-esteem, and perceived control to determine any significant prediction. All the variables accounted for 7% of the variance in COVID protocol adherence (R^2^ = 0.07, F (3, 661) =17.29, p<0.001) with perceived control significantly predicting adherence to COVID safety protocol (B = 0.11, β=0.23, t=5.54 p<0.001).

**Conclusion:**

Results indicated that perceived control over important life events and healthy self-esteem would likely facilitate adherence to COVID safety protocols and attenuate psychological distress. Implications for further research and design of appropriate COVID coping response interventions are discussed.

**Funding:**

Internally generated

## Introduction

The 2019 COVID pandemic is a global concern. The evidence across continents suggests that it has impacted, and continues to impact, both rich and developing countries.^1^ Since the occurrence, countries worldwide have implemented measures to curb its deadly impact. In the initial wave of the pandemic, there were total or partial lockdowns of various facets of social functioning except for essential services providers like healthcare and food sellers. Ghana was no exception.

The lockdown, even partial lockdown, was psychologically^2^ and economically^3^ difficult for many. Reports of anxiety, depression and stress were not uncommon, especially among single, separated, or widowed persons and those with larger family sizes, as loss of jobs or income and economic implications were harsh realities for many.^2^

Aside from the economic impact, the psychological effects of the pandemic appear worldwide as populations show varied, yet high rates of symptoms of stress, anxiety, depression, and other forms of psychological distress, including post-traumatic stress disorders.^4^ Even though in many countries there are indications of stress, anxiety and depression, middle and lower-middle-in-come countries differ in levels of psychological impact and distress associated with the COVID experience, with countries of higher economic status showing relatively higher levels of psychological distress.^1^,^5^ The vulnerable include women, young adults, students,^4^, ^6^ urban dwellers, those with frequent exposure to social media, and persons with pre-existing physical or psychiatric illnesses.^7^

Studies have shown that prompt and stringent government response measures moderated the adverse psychological impact of the pandemic. In a review of studies from thirty-three countries around the globe, within various income brackets, it was observed that the prevalence of clinically significant depressive symptoms was significantly lower in countries wherein governments acted promptly. The moderating effect of government response remained significant even after controlling for variances in the frequency of COVID cases;^8^ suggesting that prompt government action was psychologically beneficial to countries' populations.

The Government of Ghana acted promptly by putting in stringent COVID control measures. On 15th March 2020, the Government of Ghana ordered the closure of all educational institutions. Despite the closure of schools, the Ministry of Education and the Ghana Education Service were quick to put measures in place to facilitate opportunities for continuous learning.^9^ Around the world, when government prevention control measures seemed to work in curbing the spread of the virus and in the number of deaths, some semblance of normalcy was resumed in various countries by easing restrictions. Likewise, Ghana gradually eased restrictions and reopened schools. Thus, in January 2021, tertiary institutions in Ghana were given the ‘green light’ to reopen fully. Around this time, there were reports of another deadly variant of the COVID-19 virus in other parts of the world, including Africa.

The Kwame Nkrumah University of Science and Technology (KNUST) is a tertiary institution with over sixty thousand students on its Kumasi campus. Efforts on adherence to protocols aside, the huge number of students on campus is a cause of serious concern in light of reports of the resurgence of a more insidious new variant of the COVID-19 virus with less clearly defined symptom presentation and higher death tolls.

As already noted, university students are a vulnerable population^10^,^4^,^6^ with higher levels of anxiety, depression, substance abuse and disordered eating than the general population.^11^ Students also exhibit psychological symptoms of anxiety, stress and depression.^12^ With the genesis of COVID-19 and consequent radical and constraining changes in their educational experience, the burden on the mental health of this vulnerable population appears amplified as their well-being is compromised.^13^,^14^

A recent study examining the psychological impact of COVID-19 on college students following the reopening of schools shows that in the post-lockdown period, students still exhibit signs of anxiety and depression.^15^ Profiles of at-risk students for psychological distress include being 18-24 years old, female, knowing someone infected, and being from below-average family income homes.^11^, ^15^

However, greater self-rated health status and adherence to precautionary measures have been associated with lower psychological impacts.^12^ Individuals' perception, such as perceived survival likelihood, is protective as it influences coping response.^16^ Risk perception has been associated with greater adherence to protective action recommendations.^17^ Although people from different nations (and cultures) may hold different views about adhering to protective measures, such as wearing masks, the evidence suggests that wearing a mask is more beneficial. China and Poland, for example, differ in their perceptions about (and practice of) wearing masks, with the Chinese showing stricter adherence to mask-wearing than the Polish; the evidence showed that mask-wearing was associated with better physical and mental health.^18^ Likewise, students' adherence to safety protocols (like wearing masks, hand washing and social distancing) has been associated with lower anxiety levels.^15^ Conversely, students who perceive self-immunity because of their youth are less compliant with recommended COVID protocols^19^, suggesting that students' perception plays a role in their coping response to the pandemic.

The perceived control literature consistently shows that feeling in control over events plays a critical role in people's psychological and physical wellbeing.^14^, ^20^, ^21^ Greater perceived control had been associated with positive effects on different areas of life, including positive self-concept;^22^ attenuations of stress 14, 23, 24 and healthy psychological functioning.^23^, ^25^, ^26^, ^27^, ^28^

Though control beliefs operate at the cognitive level and may not necessarily be accompanied by actual behavioural manifestation, individuals' control beliefs may inform their control strategy and coping response in a given situation. Accordingly, perceptions and control beliefs have been shown to moderate reactions and responses to the COVID pandemic.^15^, ^19^, ^29^, ^30^, ^31^

Similarly, possessing good self-esteem and self-efficacy; that is, believing self to be efficacious in effecting desired outcomes, have been found to be influential factors for predicting psychological distress during the current COVID-19 pandemic.^19^ Higher levels of self-esteem and self-efficacy correlated with lower levels of psychological distress.^23^

Significant factors influencing students' mental health status during this pandemic reflect what students do or do not do, such as engaging in healthier personal lifestyles (higher exercise frequency, lower alcohol use, better sleep quality and wearing masks) associated with lower risk of psychological problems.^15^

In Ghana, some work has been done to unearth mental health challenges associated with the COVID-19 pandemic.^32^ The present situation of huge student numbers on the KNUST main campus and the resurgence of a reportedly deadlier virus must be well understood to help develop evidence-driven and context-specific strategies and interventions to reduce the adverse psychological impacts of the pandemic among students. Efforts to recognise and address university students' mental health challenges, especially during a pandemic, are critical because of potential long-term consequences on their health and education. Therefore, this study aimed to examine and identify the psychological factors associated with students' response to the COVID pandemic to aid in formulating a best-fit situational response for them.

Based on the existing literature and the theoretical underpinnings of variables of interest, two main hypotheses were examined in this study. One, that students with higher perceptions of control or greater self-efficacy are more likely to adhere to COVID protocols. Two, that psychological distress would be greater among students with less self-efficacy and control perceptions. We expected a positive correlation between perceptions of control and adherence to COVID protocols but a negative correlation between perceptions of control and psychological distress. In effect, negative correlations were predicted between psychological distress variables and perceptions of control. Conversely, a positive correlation was predicted between psychological distress variables and COVID risky behaviours.

## Methods

### Study design

The study was a cross-sectional survey conducted among students of the Kwame Nkrumah University of Science and Technology (KNUST). A quantitative, non-experimental, correlational design examined relationships among interested variables. Representative and convenient sampling was used to invite students to complete the questionnaire in February-April, 2021.

### Setting

The KNUST is one of the public universities in Ghana, located in Kumasi in the Ashanti Region of Ghana. In January 2005, the university was organised into six colleges to facilitate greater academic and administrative autonomy. These are the College of Agriculture and Natural Resources (CANR), College of Art and Built Environment (CABE), College of Engineering (CoE), College of Humanities and Social Sciences (CoHSS), College of Health Sciences (CoHS) and College of Science (CoS). Presently, the university has a student population of over sixty thousand and a teaching staff strength of about 4,000. The university enrols students from all regions and outside Ghana, with the international student population forming close to three per cent of the student body.

### Participants and Procedure

Participants were randomly sampled with no particular efforts for even gender distribution. Using the formula of Tabachnick & Fidell,^33^ a total of 751 students participated in the study, having been solicited from classes. Those who agreed to participate were given adequate information about the study and its potential benefits to them. Verbal consent was sought, and the opportunity to withdraw in the event of refusal was given before hard copies of the questionnaire were administered. The questionnaire was also digitised (web-based) for ease of administration and completion by participants who wished to do so electronically. The brief questionnaire took less than ten minutes to complete, with no known risks and the potential benefit of greater self-awareness for participants. Anonymity was ensured, and there was no identifying information linking responses to participants in any way. Data were coded and entered into a computer program (SPSS) for analysis. In the data management and ‘cleaning’, significantly incomplete data were excluded from the analysis.

### Measures/Instruments

Self-report questionnaire was used to collect data for the study. The first part consisted of demographic information on participants. The second part consisted of questions on adherence to COVID safety protocols, psychological functioning, self-esteem, and perceptions of control. Questions regarding the observance of COVID-19 safety protocol, such as wearing a mask, handwashing and observing social distance, were rated on a four-point Likert scale from 1- ‘Never’ to 4 – ‘Always’, with higher scores indicating greater adherence to safety protocol.

The Depression, Anxiety, and Stress Scale (DASS-21; a shorter version of the DASS-42)^34^,^35^ was used to assess psychological functioning. The DASS-21 quantitatively measures distress along the three axes of depression, anxiety and stress. It is a screening tool and not a categorical clinical diagnosis measure, consisting of twenty-one items/statements with seven each for depression, anxiety and stress. It asks respondents to indicate how much of the symptoms of depression, anxiety and stress they have experienced within a period. It is rated on a four-point Likert scale as follows:

0 - Did not apply to me at all – NEVER

1 - Applied to me to some degree, or some of the time - SOMETIMES

2 - Applied to me to a considerable degree, or a good part of the time - OFTEN

3 - Applied to me very much, or most of the time - ALMOST ALWAYS

Higher scores indicate higher symptoms. Symptom presentation is categorised as ‘normal’, ‘mild’, ‘moderate’, ‘severe’, and ‘extremely severe’ as seen in the table below.

**Table:** DASS Severity Scores

Severity	Depression	Anxiety	Stress
Normal	0–4	0–3	0–7
Mild	5–6	4–5	8–9
Moderate	7–10	6–7	8–9
Severe	11–13	8–9	13–16
Extremely Severe	14+	10+	17+

The DASS has good psychometric properties36; with good reliability coefficients for non-clinical normative population (Cronbach's alphas of 0.88 for the depression scale; 0.82 for the anxiety scale; 0.90 for the stress scale, and 0.93 for the full scale), that indicate strong internal consistency of the constructs. During the COVID-19 pandemic, the DASS-21 has been used as a valid measure in different cultures and countries.^5^, ^16^, ^37^, ^38^ In this study, a composite mean score of psychological distress was used for the analyses since diagnostic categories or impressions was not a focus.

Items used to measure perceptions of control were adapted from existing primary and secondary control scales.^39^, ^40^, ^41^ This set of control items have demonstrated good internal consistency (coefficient alphas of 0.72 for primary control and 0.79 for secondary control) in previous work. It is an eight-item questionnaire rated on a seven-point Likert scale from 1 – ‘Not at all true of me’ to 7 – ‘Very true of me’. Higher scores indicate greater individual control appraisal.

Self-esteem was measured using the Rosenberg Self-Esteem Scale, used cross-culturally and consistently with good psychometric properties.^43^,^44^ It is a ten-item scale with questions rated on a four-point Likert scale that asks respondents to rate themselves on the items from 1 – strongly disagree to 4 – strongly agree. Items that are worded in the negative are reversed scored. Higher scores indicate stronger self-esteem.

### Ethical Clearance

Ethical clearance for the study was sought, and approval was obtained (CHRPE/AP/132/21) from the Committee on Human Research, Publication and Ethics (CHRPE) of the KNUST.

## Results

Demographic information on participants is presented in Table 1. There were more female participants relative to males, with the majority aged between 15-25 years and comparatively fewer within the age range of 26-30 years and above. While most indicated they were single, others were in non-marital relationships. Very few students were married. Almost all participants were Ghanaians, with a few international students. The majority of the participants were Christians, a few Muslims, and very few others endorsed traditional or other religious denominations. Participants were predominantly from the College of Health Sciences (where research was nested), and most students were in their second or third year of undergraduate study. Regarding their residential status (while in school), most participants lived outside the university campus, with about a third on campus.

**Table 1:** Demographic characteristics of participants

Variables	N	%
**Gender**		
Male	276	37.7
Female	456	62.3
**Age**		
15–20	314	44.0
21–25	351	49.2
26–30	26	3.7
30 and above	22	3.1
**Marital Status**		
Single	556	76.4
In non-marital relationship	146	20.0
Married	26	3.6
**Nationality**		
Ghanaian	722	98.6
International	10	1.4
**Religious Group**		
Christianity	662	89.6
Islam	68	9.2
Traditional	05	0.7
Others	04	0.5
**Year**		
1	26	3.6
2	263	36.1
3	291	40.0
4	144	19.8
Graduate	4	0.5
**Colleges**		
Health Sciences	566	77.2
Humanities and Social Sciences	144	19.7
Engineering	14	1.9
Others	09	1.2
**Residential Status**		
On-Campus	228	33.0
Off-Campus	463	67.0

Relationship among the variables was examined with bi-variate correlational analysis. Table 2 shows the inter-correlations among self-esteem, psychological distress, perceived control and COVID protocol adherence. All correlations were statistically significant and in the predicted direction (see Table 2).

**Table 2:** Intercorrelations among self-esteem, perceived control, psychological distress, and COVID protocols adherence

	Variables	M	SD	N	2	3	4
**1**	Self-Esteem	3.24	0.53	745	0.40***	-0.44***	0.16***
**2**	Perceived Control	5.31	1.27	745		-0.20***	0.24***
**3**	Distress	0.70	0.57	745			-0.12***
**4**	Covid Adherence	2.95	0.53	745			

* p< .05

** p< .01

*** p< 0.001

Self-esteem significantly and positively correlated with perceived control (*r* = 0.40, *p*<0.001) and COVID adherence (*r* = 0.16, *p*<0.001); suggesting that students with higher self-esteem and greater perceived control are more likely to adhere to COVID safety protocols believing that they can effectively control important happenings in their lives, including health. However, self-esteem negatively correlated with psychological distress (*r* = -0.44 *p*<0.001), implying that as a student's self-esteem increases, the less likely it is that one would be psychologically distressed and vice versa. Likewise, greater perceived control was associated with lower psychological distress (*r* = -0.20 *p*<0.001), and greater adherence to COVID protocol (*r* = 0.24 *p*<0.001).

These findings suggest that an individual's perception of control over important things in life, and self-confidence in the efficacy of desired outcomes, the more likely the one would adhere to the COVID protocols to sustain good health. Moreover, the more one perceives self to be in control of happenings around him/her, and the less likely s/he would be psychologically distressed. This is not surprising because if one believes one can influence, control, and make choices that affect one's life, one is more likely to ‘do something’ about any situation instead of leaving things to chance or taking a victim position. For example, adherence to COVID safety protocols would be perceived as a necessary behaviour within personal control for the safety of own life. In effect, individuals who believe themselves capable and that their choices and decisions matter are less likely to be distressed. The psychologically distressed, conversely, are less likely to adhere to the COVID safety protocols, as evidenced by the significantly negative correlation between distress and COVID adherence (*r* = -0.12 *p*<0.001).

The more distressed individual is less likely to adhere to safety protocols and vice versa. Examination of the correlation of the DASS subscales with other variables was conducted to understand more clearly which of the three DASS subscales (depression, anxiety, and stress) was more associated with or underlying psychological distress. This showed the depression subscale having relatively larger coefficients with stress (*r* = 0.78, *p*<0.001), self-esteem (*r*=-.51, *p*<0.001), perceived control (*r* =-.25, p<0.001) and adherence to safety protocol (*r* =-.11, p<0.001). It seemed that apathy, lack of motivation, and anhedonia which are symptomatic of depression, would influence psychological distress more than anxiety. Therefore, it was unsurprising that students with higher levels of depression (and therefore greater psychological distress) would find it difficult to care for themselves or their health and safety.

Furthermore, to determine the variables that significantly predicted adherence to COVID safety protocols, COVID adherence was regressed on psychological distress (DASS), self-esteem and perceived control. All the variables accounted for 7% of the variance in COVID protocol adherence (R^2^ = 0.07, F (3, 741) =17.29, *p*<0.001). As seen in Table 3, only perceived control significantly predicted adherence to the COVID protocol (B = 0.09, β=0.21, t=5.48 p<0.001). The other variables did not significantly predict adherence. This implies that students who believed they had control over their lives were more likely to adhere to the COVID protocols; these students were also not likely to be psychologically distressed, as seen in Table 2.

**Table 3:** COVID protocol adherence regressed on distress, self-esteem and perceived control

	B	SE	β	t	R^2^	F
					0.07***	17.29
**Constant**	2.36	1.48		15.98		
**Distress**	-0.05	0.04	-0.05	-1.37		
**Self-Esteem**	0.05	0.04	0.05	1.13		
**Perceived** **Control**	0.09	0.02	0.21***	5.48		

* p<0.5

** p< .01

*** p<.001

## Discussion

This study sought to delineate psychological correlates of the COVID-19 response among the KNUST students since the university's reopening and to gather the information that would inform the formulation of context-specific psychological interventions for students. Variables examined included self-esteem, psychological distress (anxiety, depression, and stress), and perceptions of control about adherence to COVID-19 safety protocols.

As hypothesised and consistent with existing literature, the study's findings generally showed that robust psychological well-being could facilitate good behavioural response to COVID. Significant correlations existed between psychological indices (distress, self-esteem, perceptions of control) and adherence to COVID protocols, as shown by the inter-correlations in Table 2. Specifically, students with stronger self-esteem and greater perceptions of control over important events were less likely to be psychologically distressed and more likely to adhere to COVID protocols. In other words, these students felt ‘more in charge’ of their lives and believed that what they did or did not do impacted the outcome (what happened to them) regarding the pandemic. Conversely, those students who held lower self-esteem and lower perceptions of control were more likely to be distressed and less likely to adhere to the COVID protocols of safety.

These findings of this study are consistent with previous works that show a positive impact of perceptions of control and self-esteem for mitigation of psychological distress.^14^,^20^,^21^,^22^,^25^,^26^,^27^, ^28^ The perceived control literature, for example, consistently shows that feeling in control over important life events plays a critical role in the attenuation of stress and individuals' psychological and physical well-being. Some studies since the pandemic have shown that control beliefs, including perceived survival likelihood^16^, moderate reactions and response to the COVID pandemic. 19, 29, 30, 31 Similarly, having good self-esteem or believing self to be efficacious are an influential factor for predicting psychological distress during the current COVID-19 pandemic.^19^ In one study, higher self-esteem and self-efficacy correlated with lower psychological distress levels.^23^

In the present study, to better help us design a most efficacious psychological intervention for students, we further examined which variables were most predictive regarding adherence to COVID safety protocols. The regression analysis (see Table 3) showed that perceived control was the most significant predictive variable for adherence to safety protocols. Again, this is consistent with research on perceived control in empowering individuals' coping responses.^19^ This finding indicates that a good psychological intervention would enhance students' control beliefs for safety.

Control beliefs operate at the cognitive level and may not necessarily be accompanied by actual behavioural manifestation; however, a good control appraisal and belief in one's capability informs the choice of control strategy or coping response in any given situation. In previous studies, persons with greater perceived survival likelihood^16^ and those who believed that their actions (like mask-wearing) would be protective of them^18^ showed better physical and mental health. Therefore, an intervention that encourages students to believe that what they do (for example, wearing their masks) or do not do (for example, congregating without appropriate social distance) would impact whether they contract the virus. They must believe that the control to effect a desired change or manage a situation lies within their power, and exercising it would boost their subjective well-being (reduce distress). In other words, psycho-educational programs that consolidate students' control perceptions and help them understand the synergistic and contingent interactions between their own behavioural choices and desired outcomes (i.e. safety or avoidance of ill health) would be a step in the right direction.

The KNUST Counselling Centre and other university counselling centres need to consider psycho-educational programs that enhance students' perceptions of control. This and frequent online cognitive restructuring ‘nuggets' on student platforms that empower self-esteem and control perceptions can improve students' subjective well-being, particularly during this global pandemic.

The level of the psychological impact of the pandemic changes over time, as observed in a longitudinal study where there was a significant reduction in self-reported psychological distress (anxiety, stress, and depression) four weeks after the outbreak of the pandemic.^16^ The present research is a cross-sectional study; therefore, the observed psychological distress reflects the study period.

Other limitations include the predominance of students from the college where the study was nested (College of Health Sciences) relative to other colleges of the KNUST. We also observed more females than males in the study and is arguably attributable to the consent procedure, where participants could voluntarily opt-in or out of the study. More females volunteered to participate.

Limitations of the study notwithstanding, this research provides good insights for designing context-specific psychological response interventions for students at the KNUST. Additionally, the findings provide a basis to investigate further other moderating variables for greater adherence to COVID safety protocols aside from perceived control. For example, a follow-up study that examines students' perceptions of and willingness to receive the COVID-19 vaccination would add to our understanding of ways to combat the pandemic among students. Such a study could examine, among other things, students' perceptions and concerns about the COVID vaccine and any internalised stigma associated with receiving the vaccine.^45^ More research along these lines would be worthwhile since the COVID pandemic and consequent psychological impacts are far from over.

## Conclusion

As the COVID-19 pandemic continues to be a health concern, the safety of students on university campuses with respect to adherence to safety protocols needs attention. All must mask up and adhere to COVID safety protocols, but who will likely do so? This study examined the psychological correlates of adherence to safety protocols among students on the KNUST campus. Findings showed that perceived control and robust self-esteem influence safety behaviours and attenuate psychological distress.
